# Allyl Isothiocyanate Inhibits Actin-Dependent Intracellular Transport in *Arabidopsis thaliana*

**DOI:** 10.3390/ijms161226154

**Published:** 2015-12-07

**Authors:** Bjørnar Sporsheim, Anders Øverby, Atle Magnar Bones

**Affiliations:** Department of Biology, the Norwegian University of Science and Technology, Høgskoleringen 5, N-7491 Trondheim, Norway; bjornar.sporsheim@ntnu.no

**Keywords:** glucosinolate, sinigrin, allyl isothiocyanate, actin cytoskeleton, intracellular transport, plant defense mechanism

## Abstract

Volatile allyl isothiocyanate (AITC) derives from the biodegradation of the glucosinolate sinigrin and has been associated with growth inhibition in several plants, including the model plant *Arabidopsis thaliana*. However, the underlying cellular mechanisms of this feature remain scarcely investigated in plants. In this study, we present evidence of an AITC-induced inhibition of actin-dependent intracellular transport in *A. thaliana*. A transgenic line of *A. thaliana* expressing yellow fluorescent protein (YFP)-tagged actin filaments was used to show attenuation of actin filament movement by AITC. This appeared gradually in a time- and dose-dependent manner and resulted in actin filaments appearing close to static. Further, we employed four transgenic lines with YFP-fusion proteins labeling the Golgi apparatus, endoplasmic reticulum (ER), vacuoles and peroxisomes to demonstrate an AITC-induced inhibition of actin-dependent intracellular transport of or, in these structures, consistent with the decline in actin filament movement. Furthermore, the morphologies of actin filaments, ER and vacuoles appeared aberrant following AITC-exposure. However, AITC-treated seedlings of all transgenic lines tested displayed morphologies and intracellular movements similar to that of the corresponding untreated and control-treated plants, following overnight incubation in an AITC-absent environment, indicating that AITC-induced decline in actin-related movements is a reversible process. These findings provide novel insights into the cellular events in plant cells following exposure to AITC, which may further expose clues to the physiological significance of the glucosinolate-myrosinase system.

## 1. Introduction

**Figure 1:**
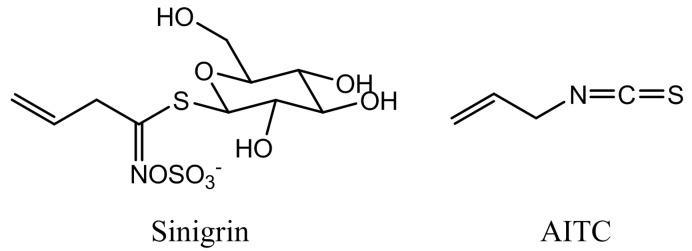
Chemical structures of sinigrin and allyl isothiocyanate (AITC).

Allyl isothiocyanate (AITC; Figure 1) is a volatile and reactive compound displaying bioactivity in a range of cell types. It is found in several plants belonging to the *Brassicaceae* family, where it is stored as the inert precursor sinigrin (Figure 1). The glucosinolate sinigrin is a substrate for the thioglucoside glucohydrolase myrosinase (EC 3.2.1.147), and these components are maintained physically separated under normal circumstances. However, when plant tissue is ruptured, e.g., by herbivores, the two components engage in contact, resulting in a release of several potentially toxic products, including AITC [1,2,3,4]. The reactivity of isothiocyanates (ITCs) has been accredited to the –N=C=S group, and this moiety of ITCs readily binds to sulfhydryl groups. Thus, proteins containing cysteine residues with exposed thiol groups serve as easily-accessible binding targets for ITCs [5]. This renders ITCs active in a vast spectrum of cell types suggesting the glucosinolate-myrosinase defense system as an important line of defense against a variety of microorganisms that pose a threat to the plant. The activity of AITC has also been previously demonstrated in plants by inhibition of growth and/or seed germination of several weeds, wheat, palmar amaranth and the model plant *Arabidopsis thaliana* [6,7,8,9]. Studies involving the effect of ITCs in plants have been especially focused on weed control for which ITCs or ITC-producing entities constitute promising agents [10,11,12,13,14]. However, the underlying mechanisms of action are scarcely investigated in plants. Recently, we found an ITC-induced disruption of microtubular filaments in *A. thaliana* as a mechanism contributing to the observed inhibited growth phenotype, as well as the potential of ITC to affect the cell cycle of *A. thaliana* [15,16]. Moreover, ITCs have been reported to induce a transcriptional upregulation of genes encoding heat shock proteins and glutathione *S*-transferases in *A. thaliana* [9,17,18]. Khokon and colleagues reported an AITC-induced stomatal closure in *A. thaliana*, hypothesizing that this could lead to the suppression of water loss and the invasion of microorganisms through stomata [19]. Based on this, it was suggested that the physiological importance of the glucosinolate-myrosinase system is likely not limited to counteracting external threats, but may also induce internal mechanisms for plant survival [19]. In addition to their role in plant physiology, ITCs have raised much attention as potent anti-carcinogenic agents. The chemopreventive potential has been shown through epidemiological studies, *in vitro* studies with mammalian cancer cell cultures and *in vivo* testing using animal models [20,21]. Using an *in vitro* approach, Mi and colleagues have highlighted the binding and modification of proteins leading to their degradation as an important event in the chemoprotective role of ITCs [22]. More specifically, tubulins were found to be bound by ITCs with the subsequent ubiquitin labelling and degradation leading to disruption of microtubules in human lung cancer cells and human gastric cancer cells [23,24]. In addition to tubulins, actin, as well as the myosin light chain have also been identified as binding targets for ITCs in these cells [5]. Like tubulins, actin proteins constitute proteins that are highly conserved among eukaryotic cell systems with high similarities in sequence and structure. In plants, myosins are divided into two main classes: myosin VIII and myosin XI, which are involved in the movement of organelles, ER remodelling and cytoplasmic streaming [25]. We therefore hypothesized an AITC-induced effect on actin filament dynamics and functionality in *A. thaliana* based on: (i) the ability of AITC to directly bind actin subunits or filaments on accessible thiol groups of cysteine residues that are highly conserved among mammalian and plant cell systems; and (ii) the secondary effect on actin filaments by disruption of microtubules [16] due to the mutual dependency for optimal functionality for actin filaments and microtubules, or by interfering with the functionality of myosins. The present study demonstrates that AITC does not induce the disintegration of actin filaments in *A. thaliana*, but reversibly inhibits the movement of filaments and actin-dependent intracellular transport of the Golgi apparatus, endoplasmic reticulum, peroxisomes and vacuoles. These data provide evidence of a novel AITC-induced response in *A. thaliana* and contribute to increased insight into the physiological role of the glucosinolate-myrosinase system *in planta*.

## 2. Results

### 2.1. AITC Reduces the Movement of Actin Filaments

**Figure 2:**
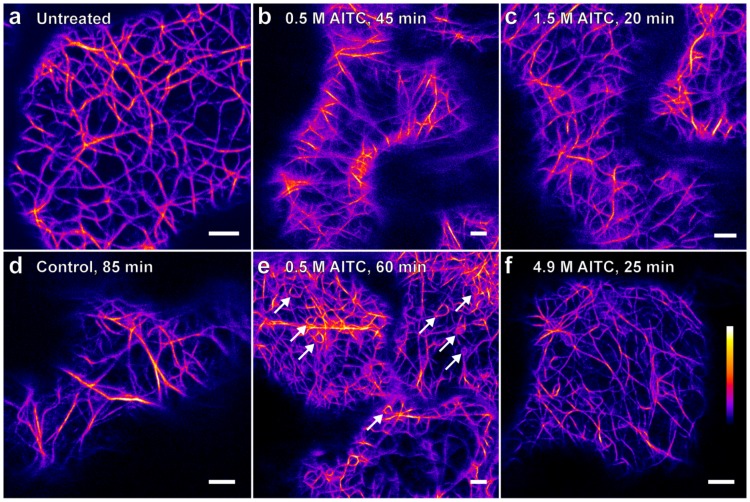
The effect of AITC on actin filaments visualized by confocal laser scanning microscopy. YFP-tagged actin filaments in seven-day-old transgenic *A. thaliana* seedlings untreated (**a**) or exposed to vehicle control (rapeseed oil) for 85 min (**d**). Exposure to the vapor-phase of 0.5 M AITC for 45 min (**b**) did not disrupt actin filaments, but introduced randomly-located curled filaments after 60 min of exposure (**e**) as indicated by the arrows. No effects on actin filaments by exposure to 1.5 M AITC for 20 min (**c**) and 4.9 M for 25 min (**f**) were observed. The ImageJ color lookup table (LUT) “Fire” was used to better visualize the actin filaments (color map displayed in (**f**)). Scale bars = 5 µm.

**Figure 3:**
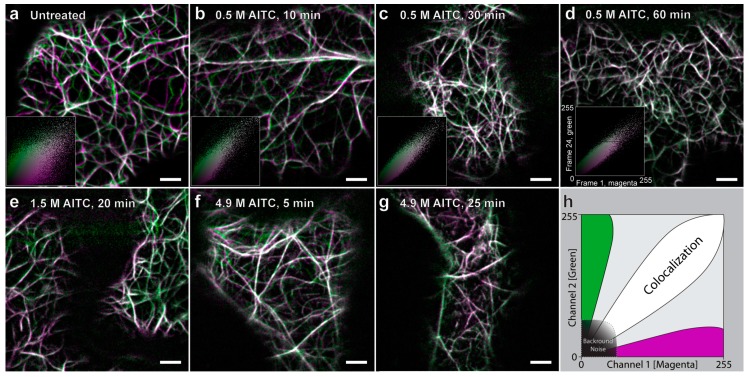
Reduction of actin filament movement by AITC visualized by confocal laser scanning microscopy. Movement of intracellular actin filaments in seven-day-old transgenic *A. thaliana* seedlings was reduced concurrently with exposure time of 0.5 M AITC varying from 10–60 min (**a**–**d**), as illustrated in the accompanying scatterplots in the bottom left corner of these images. The images are a composite of two frames of a time series, where Frame 1 is pseudocolored magenta and Frame 24 (~30 s) is pseudocolored green. The two complementary color channels combined produce white. A dose-dependent effect on actin filament movement was observed when seedlings were exposed to vapors of 1.5 M for 20 min or 4.9 M AITC for 5 and 25 min (**e**–**g**). A diagram explaining the scatterplot is shown in (**h**), where pixels that are colocalized cluster around the diagonal line from the bottom left corner to the top right corner. Pixels that show a lesser degree of colocalization are located further away from the diagonal line. Scale bars = 5 µm.

To investigate the effect of AITC on actin filaments in *A. thaliana,* a transgenic line expressing YFP-tagged actin filaments was used (Table 1; Figure 2). When actin filament movement and dynamics were investigated, a time-dependent reduction of filament movement was observed when seedlings were exposed to 0.5 M AITC for 10–30 min (Figure 3b,c). The characteristic wavy movements of actin filaments in untreated plants gradually decreased, leaving only a few of the thin filaments irregularly vibrating after 30 min of exposure. These motions were further reduced after 45 and 60 min of exposure, leaving all filaments close to static (Figure 3d). A dose-dependent effect on actin filament movement was observed in plants inspected after exposure to 1.5 M AITC for 20 min or 4.9 M AITC for 5 and 25 min (Figure 3e–g). Treatment with vapor of 1.5 M AITC for 20 min resembled the outcome of plants exposed to 0.5 M AITC for 45–60 min, whereas 4.9 M AITC strongly reduced filament movement after 5 min and rendered the filaments almost static after 25 min. We have previously found an AITC-induced disruption of microtubule filaments in *A. thaliana* in which exposure to vapor of 0.5 M AITC for 30 min led to a complete disintegration of the filaments [16]. Actin filament structures, however, appeared unchanged by exposure to vapor of 0.5 M AITC for up to 45 min (Figure 2b). When incubation time was extended to 60 min, the filament structures remained intact, but displayed randomly-located coiled actin filaments (Figure 2e). No effects on actin filament morphology were observed when exposure was repeated with increased AITC concentrations of 1.5 M for 20 min or 4.9 M for 25 min, except an increase in the number of actin coils, or ring-like structures (Figure 2c,f). The number of actin ring-like structures seemed to be loosely correlated with the concentration of AITC and the duration of exposure. Collectively, these data demonstrate that AITC did not induce disintegration of actin filaments in *A. thaliana*, but rather, fixed the actin filaments in a time- and dose-dependent manner.

**Table 1:** Transgenic *A. thaliana* lines with subcellular markers used in the present study.

Transgenic Line	YFP-Tagged Protein	Fluorescent Marker	References
*YFP-mTalin*	Talin	Actin filaments	[26]
*GOLGI-YFP*	Man1	Golgi apparatus	[27,28]
*ER-YFP*	HDEL and AtWAK2	Endoplasmic reticulum	[27,29]
*VAC-YFP*	γ-TIP	Tonoplast	[27,30]
*YFP-PER*	PTS1	Peroxisomes	[27,31]

### 2.2. Reduced Actin-Dependent Intracellular Transport by AITC

**Figure 4:**
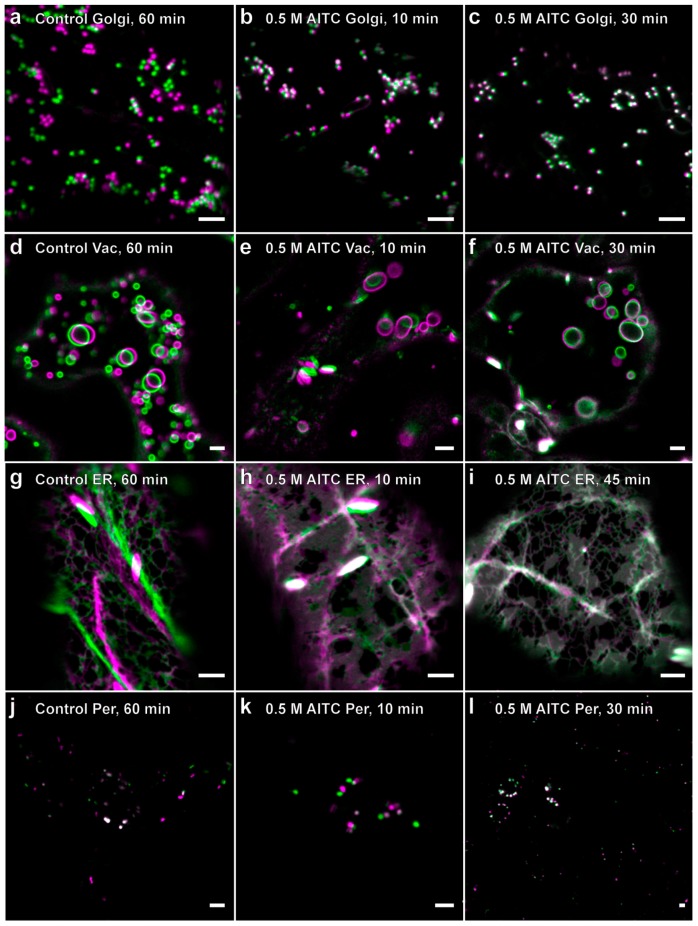
Inhibition of actin-dependent transport by AITC visualized by confocal laser scanning microscopy. Exposure of seven-day-old seedlings of transgenic lines to vapor of 0.5 M AITC for 10 and 30 (45 min for ER) min showed inhibition of intracellular movement of Golgi (**a**–**c**), vacuoles (**d**–**f**), ER (**g**–**i**) and peroxisomes (**j**–**l**). The images are a composite of two frames in a time series, where Frame 1 is pseudocolored magenta and Frame 24 (~30 s) is pseudocolored green. The two complementary color channels combined produce white. The dynamics of these organelles were slowed down to sporadic movements and vibrations when treated with 0.5 M for 10 min and further reduced when the incubation time was increased to 30 and 45 min. The treatment also changed the morphology of the ER, from a highly polygonal and reticular network organization to a broader distribution of expanded lamellae (**h**,**i**). Scale bars = 5 µm.

**Figure 5:**
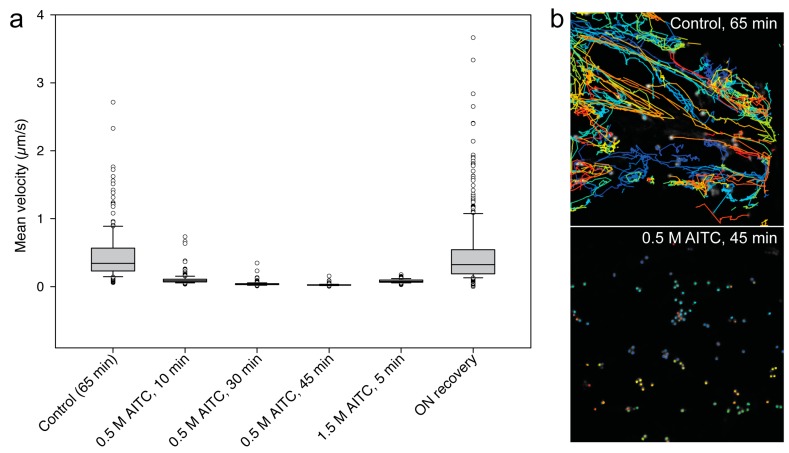
Golgi tracking and velocity measurements. The mean velocity (µm/s) is shown in a box plot (**a**), based on the results generated by detection and subsequent tracking of single Golgi bodies for different treatments (**b**). The movements of single Golgi bodies in seven-day-old transgenic *A. thaliana* seedlings were reduced in a time- and dose-dependent manner when exposed to 0.5 M AITC for 10, 30 and 45 min and 1.5 M AITC for 5 min. Seedlings treated with 0.5 M AITC for 30 min showed a full recovery of movement when incubated in an AITC-absent environment overnight. The box plot in (**a**) shows all individual outliers from each dataset, and the error bars indicate the upper 90th and the lower 10th percentile. The median is marked by a line within the box. The tracks in (**b**) are color-coded based on Track ID (individual spots).

Next, we investigated the effect of AITC on actin-dependent intracellular transport in *A. thaliana*. For this purpose, we used four transgenic lines expressing YFP-tagged proteins associated with Golgi, endoplasmic reticulum (ER), vacuoles and peroxisomes (Table 1) of which transport has previously been reported to be actin dependent [27]. Seven-day-old seedlings from each of the four lines exposed to 0.5 M AITC displayed a reduction in intracellular movement of fluorescing structures consistent with the AITC-induced reduction of actin filament movement (Figure 4). All movement was substantially slowed down 10 min after exposure, and after 30 min, movement throughout the cells could no longer be observed, leaving the structures strictly confined with movements restricted to minor sporadic vibrations (Figure 4c,f,l). These irregular oscillations were further reduced to almost static when incubation time with 0.5 M AITC was extended to 45 min (Figure 4i). Moreover, the decline in intracellular movement appeared to be dose dependent when seedlings of all four transgenic lines were subjected to vapor of 4.9 M AITC for 5 min, resulting in movements similar to those observed in transgenic seedlings exposed to 0.5 M for 30 min (data not shown). To quantify the observed decrease in movement in actin-dependent transport, we performed spot detection followed by tracking of individual Golgi bodies in seedlings treated with AITC. Our velocity measurements showed a time and dose dependency upon AITC treatment (Figure 5). In addition to the attenuated movement induced by AITC, ER displayed a change in morphology following AITC exposure (Figure 4h,i). Treatment with AITC resulted in an ER organization with expanded lamellae and less reticular network (Figure 4i). Furthermore, the highly dynamic transport routes within the ER (Figure 4g) were very sensitive to AITC and became close to absent when treated with 0.5 M AITC (Figure 4h,j). The reduced movement of Golgi was comparable to the reduction in Golgi movement when seedlings were treated with the actin inhibitor latrunculin B, with the initial fluid motion changing into more aberrant and irregular movement (data not shown). Taken together, AITC inhibited actin-dependent intracellular transport in a time- and dose-dependent manner consistently with the reduction in actin filament movement.

### 2.3. Recovery of Actin Filament Movement and Actin-Dependent Transport after AITC Exposure

**Figure 6:**
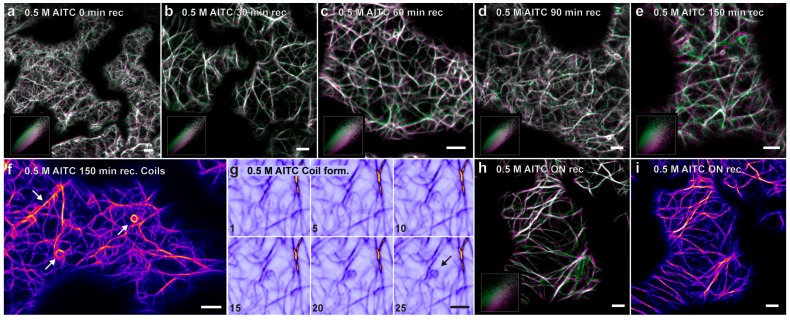
Recovery of actin filament movement and the formation of actin ring-like structures visualized by confocal laser scanning microscopy. The exposure of seven-day-old *Arabidopsis* seedlings expressing YFP-tagged actin to vapor of 0.5 M AITC for 30 min displayed a slow, but gradual regaining of dynamics from 0–150 min recovery (**a**–**e**), as illustrated in the accompanying scatterplots in the bottom left corner of these images. After an overnight recovery period, the actin filament movement appeared similar to that in plants treated with vehicle control or untreated plants (**h**,**i**). In the course of the recovery an increase in the number of actin ring-like structures was observed (indicated by white arrows in **f**). A ring formation process is shown in (**g**), where the number in the bottom left corner indicates the frame number in a time series, at a frame interval of 0.66 s. The images (**a**–**e**,**h**) are a composite of two frames in a time series, where Frame 1 is pseudocolored magenta and Frame 24 (~30 s) is pseudocolored green. The two complementary color channels combined produce white. Scale bars = 5 µm.

Finally, the ability of the attenuated movements of actin filaments and actin-dependent transport to recover from AITC exposure was assessed. In these experiments, all seedlings were exposed to 0.5 M AITC for 30 min, previously shown to be sufficient for a complete inhibition of actin-dependent intracellular transport (Figure 4), as well as strongly reducing the movement of actin filaments, leaving only the thin filaments vibrating (Figure 3c). Immediately following treatment, plants were transferred to a fresh medium free of AITC for recovery. Vehicle-treated plants were also transferred to fresh medium to balance out the effect the transfer process might have caused. Cells of transgenic seedlings with fluorescent actin filaments displayed a gradual recovery of transport over a 2.5-h incubation period initiated by random vibrations followed by a limited degree of undulating movements similar to those seen in untreated or control-treated seedlings (Figure 6a–e). However, in this recovery period, an increased number of coiled actin filament structures was observed concurrently with increased actin filament movement (Figure 6f). These actin coils were similar to those observed after 60-min exposure to 0.5 M, as seen from Figure 2e. A time-lapse sequence at 90 min of recovery demonstrates this structure being formed by an actin filament moving freely in seemingly random directions with the free end joining a nearby existing filament, resulting in a coiled structure (Figure 6g). When seedlings were allowed to incubate overnight, the array of actin filaments appeared normal regarding both movement and morphology, which indicated a full recovery from the attenuated movements and aberrant structures of actin filaments (Figure 6h,i). No apparent increase in movement of these structures could be seen for up to 2 h after stopping the ITC treatment when analyzing the recovery of actin-dependent transport in transgenic lines with fluorescent markers on Golgi, vacuoles, ER and peroxisomes (data not shown). A full recovery to the native state of transport of these structures was seen when plants were examined following an overnight incubation in AITC-free conditions, exemplified by velocity measurements of tracked Golgi bodies over time (Figure 5). The change in ER morphology induced by AITC also returned to normal appearance following overnight incubation in the absence of AITC (data not shown). These data collectively show that AITC-induced attenuations of actin filament movement and actin-dependent transport, as well as abnormal morphologies of actin filaments, ER and vacuoles constitute reversible cellular effects.

## 3. Discussion

AITC has been associated with bioactivity in several plants, including *A. thaliana* [8,9,32,33]. This activity has been reported as growth inhibition of whole seedlings or as a suppressive effect on seed germination [9,34]. The interference with cell growth has also been reported in microorganisms and collectively suggests that AITC is a defense-related compound acting towards external threats to plant growth and/or survival [35]. However, the notion of a role for AITC beyond weaponry is beginning to emerge, as the volatile phytochemical may also be important in a feedback response to alert the producing plant itself or other plants in the same population to potential threats against survival. This was supported by Khokon *et al.* (2011), who reported that AITC induces stomatal closure, thus hypothesizing that AITC could protect the producing plant itself from water loss and intrusion of potential pathogens [19]. For either fate, in order to meet full comprehension of the role of glucosinolate-myrosinase system *in vivo*, experiments that aim at elucidating the cellular effects of ITCs in plants should be undertaken. In a recent study, we found a dose-dependent inhibition of *A. thaliana* growth by AITC and further demonstrated that AITC disrupts microtubules in a time- and dose-dependent manner [16]. In the present study, we employed transgenic plants expressing YFP fusion proteins tagging either actin filaments, the Golgi apparatus, ER, vacuoles or peroxisomes to demonstrate that AITC inhibited actin-dependent intracellular transport in *A. thaliana* in a time- and dose-dependent manner. This decline appeared to be reversible when seedlings first exposed to vapor of AITC were incubated overnight in an AITC-free environment, leading to structural morphologies and intracellular movements similar to those observed in untreated or control-treated plants.

The selected doses of AITC in the present study were based on a previous report by our group, which demonstrated that exposure of the gas phase of AITC up to 3.4 M for 1 h was not lethal to *A. thaliana* seedlings [16]. When exposed to the gas phase of 4.9 M AITC for 1 h, lethality was observed. The doses used in the present study were therefore selected in order to avoid subjecting the seedlings to a lethal treatment. Based on the rapid secretion of ITCs upon damage in plant tissue, it is expected that an exposure in nature would be local with fluctuating concentrations over a limited amount of time. The present study therefore applied a gas phase exposure in order to mimic nature as closely as possible. However, the relevant physiological dose of ITCs expelled by plants in nature has not to our knowledge been reported. Khokon *et al.* reported that exposure of 10–100 µM of AITC in a liquid solution to leaves of *A. thaliana* induced a stomatal closure, presumably a part of a physiological defensive response [19]. In the present study, the gas phase of 0.5–4.9 M of AITC was employed. Although the gas phases were not analyzed for AITC concentrations, a theoretical calculation based on the mol AITC added to the chamber, the volume of the chamber and the vapor pressure of AITC (493 Pa) resulted in 17 µM AITC in the gas phase if 0.5 M AITC was used (assuming a saturated gas phase), suggesting that the current applied doses are in the range of that of the previous report. However, future studies should be undertaken in order to clarify the physiological relevant dose of ITC to which plants are exposed.

Actin proteins are highly conserved among eukaryotes despite the different number of isoforms, particularly between plants and metazoans. The actin gene family in *A. thaliana* consists of 10 members and encodes six divergent subclasses of actin that are differentially expressed specifically in different tissues [36]. From the transcripts representing the subclass of actins encoded by *ACT2* and *ACT8*, *ACT2* is strongly expressed in nearly all vegetative tissues in young, juvenile and mature plants, and it is likely to encode the dominating isoform present in *A. thaliana* [36]. These isoforms have four of five highly-conserved cysteine residues found in the mammalian isoform of actin [37]. Mi *et al.* reported actin as a binding target for ITCs in lung cancer cells [5]. Although the binding site was not identified, previous studies have revealed that the cysteine residue Cys374 in actin is susceptible to oxidation by *tert*-butyl hydroperoxide, sensitive to oxidative stress, and it might be covalently modified by *N*-ethylmaleimide, which blocks the inhibitory effects of profilin on actin polymerization [38,39,40]. This cysteine residue is also a constituent of plant actin and might represent an accessible target point for ITCs [37]. The natural issue raised in this context is whether the binding of ITCs to actin proteins or filaments is associated with a physiological response in *A. thaliana*. Actin constitutes more than merely a dynamic endoskeleton that provides mechanical support in eukaryotic cells. In addition to being a central determinant of intracellular transport in plant cells, the actin cytoskeleton also plays an essential role in signaling and may change drastically in response to a number of abiotic and biotic stimuli [41]. Furthermore, actin dynamics have been reported as crucial for stomatal movement [42]. It is known that certain phytopathogens induce the closure of stomata in a plant as a part of its pathogen- and microbe-associated molecular pattern (PAMP/MAMP)-triggered immunity. Bacterial and fungal virulence factors and toxins have been shown to counteract stomatal closure, such as coronatine produced by *Pseudomonas syringae* pv. tomato (Pst) DC3000 and fusicoccin produced by *Fusicoccum amygdali* [43,44]. In the present study, we showed that AITC inhibits the dynamic movement of the actin cytoskeleton in live *Arabidopsis* tissue, which makes it tempting to speculate that binding to actin by AITC could somehow reduce, or even neutralize, the effect exerted by the above-mentioned toxins by interfering with the signaling cascade that promotes aperture opening. Supporting this, Eun *et al.* have shown that treatment of *Commelina communis* with phalloidin, which binds to F-actin and prevents depolymerization, delays the fusicoccin-induced stomatal opening process [45]. Guard cells treated with cytochalasin D, which promotes disruption of actin filaments, has been shown to cause partial opening of stomata [46], which further suggests that stabilizing the microfilaments might restrict stomatal opening. Another study showed that actin-depolymerizing factor (ADF) was upregulated in response to the root-knot nematode *Meloidogyne incognita* infection in *A. thaliana* [47]. By downregulating one ADF isotype, they inhibited nematode proliferation, caused by a net stabilization of actin filaments [47]. As AITC has previously been reported to induce closing of the stomata in *A. thaliana*, an interesting approach would be to observe the effect on stomata when plants were treated with the phytotoxin coronatine, which induces opening of the stomata, combined with AITC to counteract.

We recently reported that AITC depletes glutathione (GSH) content and increases reactive oxygen species (ROS) level in the roots in *A. thaliana* [18]. Increased ROS production was also shown in leaves when *A. thaliana* was treated with AITC [19,48]. As reviewed, binding up accessible cysteine residues in actin (Cys374) during oxidative stress may prevent the formation of irreversible bonds and, thus, preserve actin filaments [39]. This *S*-thiolation phenomenon is a rapid and reversible process that may also be involved in the protective role of AITC. Spontaneous binding to other proteins with accessible cysteine residues unrelated to *S*-thiolation is also a likely scenario to occur upon cell entry of AITC. Myosin proteins are essential for the movement dynamics of actin filaments, as well as the movement of organelles along the filaments [25,49]. Therefore, we cannot rule out the possibility of ITC interfering with the functions of the myosins causing the aberrated actin filament dynamics observed in our studies.

Our data clearly demonstrated an increase in actin coil abundance upon AITC treatment in epidermal cells. Subsequent to treatment, when plants were given the opportunity to recover in an AITC-free environment, a temporary increase in the number of ring-like actin structures accordingly with a gradual increase in actin dynamics was observed. After overnight recovery, these structures were as rarely observed as in untreated or control-treated plants. Collectively, this might indicate different mechanisms or routes leading to coil formation, during the reduction in actin dynamics and in connection with the resumption of filament mobility in the recovery phase after treatment, although we cannot rule out that the latter route is simply a late consequence of the initial treatment. Similar actin structures have previously been reported in different organisms using a variety of fluorescent probes and visualization methods [50,51,52,53], and Smertenko *et al.* proposed the term “actin quoit-like organelles” or “acquosomes” for these structures. Although the underlying molecular mechanisms of acquosome formation and its physiological role are poorly understood, various hypotheses exist. Evidence has suggested that actin coils constitute a storage form for excess actin, which might function as a buffer to supply the need for rapid cytoskeleton reorganization [52]. Apparently, these shapes form either by actin coiling or by circularization of straight filaments and are most likely dependent on myosin and motile actin filaments [50,52,53]. There is presumably a correlation between the number of acquosomes and the physiological condition of the cell, as heat shock and wound responses have been shown to induce the formation of these structures [52,54]. These responses are also linked with AITC exposure and/or production, suggesting another defense mechanism influenced by AITC.

It has been shown that AITC limits plant growth and can even be lethal to plants if administered at high doses [9,16]. In the present study, we infrequently observed epidermal cells that apparently had undergone programmed cell death (PCD) following treatment with AITC at concentrations above 1.5 M for more than 20 min (observed as homogeneous fluorescence within the cell cytoplasm; data not shown). Even though there is the potential of an AITC-induced PCD in cells of *A. thaliana*, it is unlikely that the AITC-induced reduction in actin dynamics is caused by PCD, as the actin cytoskeleton usually is depolymerized or reorganized in response to PCD [55]. On a related note regarding other potential secondary effects caused by AITC, it has been shown that depletion of ATP in renal cells results in actin disruption and aggregation [56,57]. Moreover, Smertenko *et al.* reported that actin coil formation is dependent on the myosin ATPase activity, which further supports that the observations in the present study are not due to ATP depletion [55], although future studies should aim at clarifying this aspect during ITC exposure of plants.

## 4. Experimental Section

### 4.1. Chemicals

Rapeseed oil was purchased at a local supermarket. AITC (purity > 95%) and Murashige–Skoog basal salt (MS-salt) were purchased from Sigma, Oslo, Norway.

### 4.2. Plant Growth and AITC Treatment in the Vapor Phase

Seeds surface-sterilized with chlorine and ethanol were sown on half-strength Murashige and Skoog (MS) agar medium (MS-salt, 2.15 g/L; sucrose, 20 g/L; agar 6 g/L; pH 5.7) and stratified for 2 days at 4 °C in the absence of light. Seeds were then allowed to germinate in a 16-h day/8-h night cycle at room temperature. For ITC treatment, seedlings grown on MS-agar medium in a 9 cm diameter petri-dish were used. After removing the lid of the 9 cm petri-dish, the petri-dish was placed inside a closed 14 cm diameter petri-dish containing a filter onto which 200 µL of the AITC solutions were added, creating an artificial gas chamber. AITC was diluted with rapeseed oil to the concentrations indicated in the text, leading to exposure of the plants by AITC in the vapor phase. For the recovery tests, plants exposed to AITC were immediately transferred to fresh MS-agar media, and roots were covered by a droplet of 0.1% agarose (*w*/*v*) to avoid desiccation.

### 4.3. Molecular Imaging

For live cell imaging, transgenic *Arabidopsis thaliana* plants expressing the following established fluorescent fusion proteins under control of the CaMV35S promoter were used. Actin was visualized by yellow fluorescent protein (YFP) fused to the actin-binding domain of mouse talin (mTalin) [27]. The Golgi apparatus was visualized by yellow fluorescent protein (YFP) fused to the first 49 amino acids of soybean (*Glycine max*) α-1,2-mannosidase I (Man1) (G-yk; CS16255) [26,28]. Endoplasmic reticulum was visualized by YFP fused to a synthetic oligonucleotide encoding the ER retention signal HDEL (at C-terminus) and the signal peptide of AtWAK2 (*A. thaliana* wall-associated kinase 2; at the N-terminus) (ER-yk, CS16252) [26,29]. Tonoplast was visualized by YFP fused to the C-terminus of γ-TIP, an aquaporin of the vacuolar membrane (vac-yk; CS16258) [26,30]. Peroxisomes were visualized by YFP fused to the peroxisomal targeting signal 1 (PTS1, Ser-Lys-Leu) (px-yk; CS16261) [26,31]. Seeds of the transgenic *Arabidopsis thaliana* plants expressing the fluorescent markers for the Golgi apparatus, Endoplasmic reticulum (ER), tonoplast and peroxisomes were obtained from Nottingham Arabidopsis Stock Centre (NASC).

### 4.4. Confocal Laser Scanning Microscopy

All plants stably expressing YFP markers were imaged on a Leica TCS SP5 system attached to a DMI 6000 CS inverted microscope, equipped with an HCX PL APO CS 63X/1.2 NA water objective (Leica Microsystems, Mannheim, Germany). Images were captured using LAS AF (Leica) software (Version 2.5.1.6757; Leica Microsystems, Mannheim, Germany). The yellow fluorescent fusion proteins were excited by the 514-nm argon laser line, and the fluorescence emission was detected in the spectral range 520–570 nm with a pinhole corresponding to 1 airy unit, at a 12-bit depth. Only epidermal cells at the adaxial leaf surface of the cotyledons were imaged during this study. Representative images of 2–4 biological replicates are shown.

### 4.5. Image Processing

Image processing was performed using the software ImageJ [58]. To produce the color scatter plots (in Figure 3 and Figure 6), a 3 × 3 mean filter was applied to the images before they were subjected to qualitative colocalization analysis using the “Intensity Colocalization Analysis” [59]. Frame 1 (time point 0 s) was assigned the pseudocolor magenta, and Frame 24 (time point 30.245 s) was assigned the pseudocolor green. The two complementary color channels combined produce white. A subset of the merged images (in Figure 3, Figure 4 and Figure 6) was manually registered to compensate for unwanted lateral drift of the sample during imaging. The single images of the time series in Figure 6g were subjected to a 3 × 3 mean filter and a Kalman filter for enhanced visualization.

### 4.6. Golgi Detection and Tracking

Automated detection and tracking of Golgi bodies was performed with the plugin TrackMate in Fiji [60]. A minimum of 60 Golgi bodies was detected and tracked for more than 78 s (corresponding to a minimum of 57 consecutive images) in each dataset shown in Figure 5. In order to remove any unwanted drift, the datasets were preprocessed using the ImageJ plugin Template Matching before tracking.

## 5. Conclusions

Our data demonstrate the reversible decline in the actin dynamics and, consequently, the actin-dependent intracellular transport in *A. thaliana* exposed to AITC. This novel mechanism may be linked to one or several defense-related mechanisms in *A. thaliana* and further supports the role of ITCs in triggering intracellular defense mechanisms, as well as acting against external threats. Our data provide insight into the physiological role of the glucosinolate-myrosinase system *in vivo*.
